# Diagnostic Potential of Recombinant scFv Antibodies Generated Against Hemagglutinin Protein of Influenza A Virus

**DOI:** 10.3389/fimmu.2015.00440

**Published:** 2015-09-02

**Authors:** Roopali Rajput, Gaurav Sharma, Varsha Rawat, Anju Gautam, Binod Kumar, B. Pattnaik, H. K. Pradhan, Madhu Khanna

**Affiliations:** ^1^Department of Respiratory Virology, Vallabhbhai Patel Chest Institute, University of Delhi, Delhi, India; ^2^Project Directorate on Foot and Mouth Disease, Indian Veterinary Research Institute Campus, Mukteswar, India; ^3^Former National Consultant (Avian Influenza), WHO-India Office, Bharatiya Kala Kendra, New Delhi, India

**Keywords:** influenza, recombinant, antibodies, scFv, HA, phage display, ELISA, diagnosis

## Abstract

Human influenza A viruses have been the cause of enormous socio-economic losses worldwide. In order to combat such a notorious pathogen, hemagglutinin protein (HA) has been a preferred target for generation of neutralizing-antibodies as potent therapeutic/diagnostic agents. In the present study, recombinant anti-HA single chain variable fragment antibodies were constructed using the phage-display technology to aid in diagnosis and treatment of human influenza A virus infections. Spleen cells of mice hyper-immunized with A/New Caledonia/20/99 (H1N1) virus were used as the source for recombinant antibody (rAb) production. The antigen-binding phages were quantified after six rounds of bio-panning against A/New Caledonia/20/99 (H1N1), A/California/07/2009 (H1N1)-like, or A/Udorn/307/72(H3N2) viruses. The maximum phage yield was for the A/New Caledonia/20/99 (H1N1), however, considerable cross-reactivity was observed for the other virus strains as well. The HA-specific polyclonal rAb preparation was subjected to selection of single clones for identification of high reactive relatively conserved epitopes. The high-affinity rAbs were tested against certain known conserved HA epitopes by peptide ELISA. Three recombinant mAbs showed reactivity with both the H1N1 strains and one (C5) showed binding with all the three viral strains. The C5 antibody was thus used for development of an ELISA test for diagnosis of influenza virus infection. Based on the sample size in the current analysis, the ELISA test demonstrated 83.9% sensitivity and 100% specificity. Thus, the ELISA, developed in our study, may prove as a cheaper alternative to the presently used real time RT–PCR test for detection of human influenza A viruses in clinical specimens, which will be beneficial, especially in the developing countries.

## Introduction

Flu is a respiratory illness, caused by influenza virus, with annual global attack rate of 5–10% in adults and 20–30% in children, causing significant levels of illness, hospitalization, and death (1). Influenza virus is an RNA virus with immunogenic surface receptors, hemagglutinin (HA), and neuraminidase (NA). Error-prone RNA dependent RNA polymerase (2) and segmented genome enable influenza viruses to undergo antigenic shifts (major) and antigenic drifts (minor), which cause changes in the surface glycoproteins, HA and NA, and permit the virus to evade the adaptive immune response of the host cells. Such phenomena back the threat posed by influenza viruses for occurrence of frequent epidemics and pandemics (3), in spite of the available antivirals for treatment (4) or the vaccines for prevention and control of the disease (5). In view of such emerging and re-emerging outbreaks, timely diagnosis of the influenza virus infections in humans is essential. The developing countries, where limited resources are available, are in urgent requirement of alternate strategies to the expensive molecular real time RT–PCR test (6), which is currently used for diagnosis of flu. The cheaper alternative could highly minimize the loss of resources caused by frequent occurrence of the influenza virus infections. In the wake of such an issue, the present study was undertaken to develop recombinant antibodies (rAbs) against the HA antigen of human influenza A virus by phage-display technique for their subsequent use in diagnosis and/or therapy. Antibodies play important role in the course of natural protection against influenza virus infections; the HA antigen being the major target (7, 8).

Monoclonal antibodies (mAbs), since their advent in 1975 by Kohler and Milstein, using hybridoma technology, have proved to be potential diagnostic molecules (9–11). However, due to the well-known limitations of the conventional hybridoma technique, development of recombinant monoclonal antibodies (rAbs) by newer and more efficient molecular methods is preferred. Such rAbs have contributed immensely in medical and pharmaceutical research, cancer therapy, and diagnosis and treatment of infectious diseases (12–15). A major advancement in the production of rAbs has been the invention of phage-display technology, where high affinity and high specificity antibodies can be developed (16) against the antigen of interest.

In the present study, we developed recombinant single chain variable fragment (scFv) antibodies against the HA antigen of A/New Caledonea/20/99 (H1N1) virus. Anti-HA influenza mAbs have been developed for various applications, however, most of them are either hybridoma-based (17–20) or against HPAI H5N1 viruses (21, 22). The anti-HA rAbs produced in the current work showed cross-reactivity against the A/California/07/2009 (H1N1)-like or/Udorn/307/72(H3N2) viruses and helped in the development of a diagnostic ELISA test with 83.9% sensitivity and 100% specificity. The therapeutic efficacy of the one heterosubtypic rAb (C5) is being evaluated in our laboratory, which has shown promising results so far (data not shown). Further assessment of the anti-HA influenza rAb developed in the present work would help to validate its efficiency as potential diagnostic and therapeutic agent.

## Materials and Methods

### Hyperimmunization of mice

Five 6–11 weeks old Balb/c mice were immunized via intra-muscular route with 1:128 HA titer of influenza A/New Caledonia/20/99(H1N1) virus, in a final volume of 100 μl, made up with 1× PBS (23). The virus administration was done on 20th, 35th, 42nd, and 51st days post first immunization. A day before each immunization, the mice sera were collected and tested for detection of anti-HA antibodies by indirect Enzyme Linked Immunosorbent Assay (ELISA) against the HA antigen of the influenza A/New Caledonia/20/99 (H1N1) virus. Permission for animal experiments was obtained by the Institutional Animal Ethics Committee (IAEC), which is registered under Committee for the Purpose of Control and Supervision of Experiments on Animals (CPCSEA), Government of India.

### Indirect ELISA

The HA antigen of influenza A/New Caledonia/20/99(H1N1) virus diluted in NaHCO_3_ coating buffer pH 9.6 was coated onto ELISA wells by incubation at 4°C for overnight. The wells were washed twice with PBST (1× PBS containing 0.5% Tween 20), and blocked with 1% BSA in PBS for 1 h at room temperature (RT). The wells were washed four times with PBST and then incubated with different dilutions of the immunized mice sera as primary antibody at RT for 2 h. The wells were washed vigorously with 1× PBST five times and incubated with HRP-conjugated rabbit anti-mouse antibody (1:1000 dilution in PBS) at RT for 1 h 30 min. The unbound secondary antibody was removed from the wells by 5–7 washings with PBST followed by addition of 50 μl of the TMB substrate solution and incubation for 25 min at RT. The reaction was stopped by the addition of 1M sulfuric acid and the absorbance was measured at 450 nm in the ELISA reader (EL_X_800_MS_, Biotek Instruments, Inc., Winooski, VT, USA).

### Collection of spleen and preparation of cDNA template

After establishment of sufficient immune response against the virus strain, on 53th day, two of the five mice were given intranasal instillation with influenza A/New Caledonia/20/99 (H1N1) virus, followed by aseptic dissection on the 55th day for collection of spleen. Following RBC lysis, the spleen cells were re-suspended in 1 ml of RPMI medium and subjected to live cell count estimation using Neubauer’s Hemocytometer. Aliquots of approximately 3.5 × 10^6^ cells were made and re-suspended in five volumes of RNA*later* (Sigma-Aldrich). After overnight incubation at 4°C, one aliquot of the cell suspension was subjected to total cellular RNA isolation using RNeasy Mini Kit (Qiagen) and thereafter total mRNA isolation using the Oligotex mRNA Mini Kit (Qiagen) as per the manufacturer’s instructions. Synthesis of cDNA was done using M-MuLV Reverse transcriptase enzyme. The reaction mix (final volume of 25 μl) consisted of 10 μl of the isolated mRNA, 800 μM dNTP mix, 200 U of M-MuLV enzyme, 0.2 μl RNase inhibitor, 2 μl oligo-dT primer, and 1× M-MuLV RT buffer. The reaction was set-up at 42°C for 2 h followed by heat inactivation of enzyme at 94°C for 5 min.

### Amplification and cloning of scFv gene repertoire

The variable light (V_L_) and variable heavy chain (V_H_) genes were amplified from the cDNA using degenerate mouse IgG primer set (Cat. No. F2010, Progen Biotechnik, GmBH, Germany), consisting of 11 degenerate forward primers for either V_H_ chain gene or V_L_ chain gene amplification. Initially, a 10 μl reaction mix was set-up using primer set I. The reaction consisted of 2.5 μM of each primer, 1× PCR buffer, 2.5 U of Hot Star *Taq*DNA polymerase (Qiagen), 0.5 mM of the dNTP mix, 2 μl of cDNA, and 2 mM of MgCl_2_. Initial denaturation was carried out at 94°C for 15 min followed by 35 cycles of denaturation (94°C for 30 s), annealing (55°C for 30 s), and extension (72°C for 30 s), and a final extension at 72°C for 10 min. The reaction was finally held at 4°C and the PCR products were resolved on 1.5% agarose gel. Second set of PCR reactions were carried out, using the corresponding Set-II forward primers, which showed amplification in the Set-I reaction. The second set of PCR was performed for introduction of restriction sites *Nco*I (5′) and *Hin*dIII (3′) to the amplified V_H_ gene fragments and *Mlu*I (5′) and *Not*I (3′) to the amplified V_L_ gene products. The Set-II reactions were put up as described for the Set-I. After analysis on the agarose gel, DNA bands from positive PCR reactions were purified and pooled for cloning in the pSEX81 phagemid vector.

The purified V_L_ gene pool was digested with *Mlu*I and *Not*I restriction enzymes (Fermentas) in appropriate buffer and ligated into 50 ng of the digested pSEX81 vector at 1:3 vector: insert molar ratio using 0.4 Weiss Units of T4 DNA ligase at 4°C overnight incubation. The ligated products were transformed into ultra-competent cells XL1-Blue strain of *E. coli* and plated onto ampicillin (100 μg/ml) supplemented nutrient agar plates followed by overnight incubation at 37°C. The colonies obtained over the agar plates were scraped and propagated in LB/amp (100 μg/ml) medium and subjected to phagemid isolation for restriction analysis with *Mlu*I and *Not*I REs, for confirmation of the V_L_ gene cloning in the vector.

The pSEX81-V_L_ vector was further digested with *Hin*dIII and *Nco*I R.E.s (Fermentas) in appropriate buffer and insertion of the RE digested pooled V_H_ gene DNA at 1:3 vector: insert molar ligation ratio using 0.4 Weiss Units of T4 DNA ligase in an overnight reaction at 4°C. The ligation mix was transformed and the resulting colonies were scraped and propagated for restriction analysis by *Nco*I and *Not*I REs to confirm the cloning of the complete scFv cassette in the pSEX81 vector.

### Construction of mouse recombinant phage-scFv library

#### Phage Rescue

The pSEX81-scFv transformed *E. coli* XL1-Blue cells were grown overnight in 10 ml SOB–GAT medium (SOB broth supplemented with 100 mM glucose, 100 μg/ml ampicillin and 10 μg/ml tetracycline) at 37°C with shaking at 200 rpm. The overnight culture was inoculated at 1:100 dilution in SOB–GAT medium, incubated at 37°C with shaking at 180 rpm and monitored every hour for bacterial growth till an OD_600_ of 0.3 was obtained. The lyophilized hyperphage M13K07ΔPIII (Progen Biotechnik Cat. No. PRHYPE) was re-constituted in 2 ml of the autoclaved milliQ water just before use, as per the manufacturer’s instructions and added to the log phase cells at an MOI of 20 (Multiplicity of Infection) representing the average number of phages per bacteria was calculated by using the following formula:

$$\text{MOI }=\;\frac{\text{No. of phage (ml added }\times \text{plaque forming units/ml)}}{\text{No. of bacteria added}}$$

The hyperphage-infected culture was incubated at 37°C without shaking for 30 min and then shaking at 260 rpm for 30–45 min. The cells were harvested by centrifugation at 3,000 rpm for 10 min at RT and re-suspended in equal volume of pre-warmed SOB–AKT medium (SOB broth supplemented with 100 μg/ml ampicillin, 50 μg/ml kanamycin and 10 μg/ml tetracycline). The cultures were incubated overnight at 34°C with shaking at 220 rpm (24).

#### Phage Precipitation

The overnight culture was centrifuged at 3,000 rpm for 15 min at room temperature and the supernatant transferred in a fresh tube. The phage particles in the supernatant were precipitated by addition of one-fifth volume of 20% PEG, 2.5M NaCl and incubating on ice for 1 h, followed by centrifugation at 4,000 rpm/4°C for 40 min. The pellet was re-suspended in phage dilution buffer (10 mM Tris–HCl pH 7.5, 20 mM NaCl, 2 mM EDTA) and stored at 4°C until further use (24).

### Phage quantification ELISA

For optimization, two-fold dilutions of the rescued phages were prepared in coating buffer (sodium bicarbonate buffer pH 9.6), coated onto ELISA wells in duplicate and incubated at 4°C overnight. After incubation at 37°C for 15–20 min, the wells were washed thrice with PBS and blocked for 1½ h with 2% skimmed milk in PBS (SMP) at room temperature. The wells were washed three times with PBS followed by addition of different dilutions of anti-M13 mouse monoclonal antibody B62-FE2 against PVIII coat protein of the M13 phage. After 1½ h incubation, the wells were washed three times with PBS and incubated with anti-mouse-HRP conjugated antibody (SIGMA, affinity purified antibody) 1:1000 diluted in SMP, for 1 h, followed by washing with PBS with 1–2 min hold per wash and incubation with 50 μl of substrate solution (5 mg OPD, 4 μl H_2_O_2_ in phosphate-citrate buffer) at 37°C for 15 min. The absorbance was measured at 492 nm; reference wavelength being 620 nm (Magellan V 7.1). All the incubation steps were done at RT unless individually mentioned (25).

### Bio-panning

Bio-panning was optimized using protocols which differed in the choice of surface area, and type of elution buffer. The surface area tested included 96-well microtiter plate, immunotube and six-well tissue culture plate, while the elution buffers included 0.1M HCl (adjusted to pH 2.2 with glycine), trypsin (1, 2, and 5 μg/ml) and 100 mM triethylamine (pH 11.0).The optimized procedure, finally preferred for selection of HA-specific recombinant phages is as follows. A MaxiSorp Immuno Tube (Nunc, Thermo Fisher Scientific, USA) was coated with influenza A/New Caledonia/20/99 virus (1:128 HA titer) diluted in a total volume of 4 ml coating buffer [0.2M Na_2_CO_3_⋅NaHCO_3_ (pH 9.6)], and kept at 4°C overnight followed by washing with PBS and then incubation with blocking solution (2% SMP) at 37°C/1 h. The tube was washed thrice with PBS and incubated with the rescued phage, vol 1 ml, at room temperature for 1 h with shaking at 35 rpm and then without shaking for 45 min. After washing 10 times each with PBST (PBS with 0.1% Tween 20) and PBS, the antigen-specific phage particles were eluted by incubation with 1 ml of 100 mM triethylamine (Sigma) for maximum 10 min. The eluted phage was aspirated into a tube containing 0.5 ml of Tris–HCl pH 7.5, for neutralization of the eluted phage (26). The panning procedure was repeated six times for selection of HA-specific scFv antibody-phages with stringency of washing increased for each round. Total phage yield (in triplicates) was assessed after each round of bio-panning by ELISA.

### Assessment of cross-reactivity

The phages precipitated after the last two rounds of bio-panning were also assessed (in duplicates) for reactivity against the A/California/07/2009 (H1N1)-like or A/Udorn/307/72(H3N2) viruses. After the sixth round of bio-panning, 56 phage clones were randomly selected and underwent small-scale phage rescue as previously described (24). The precipitated phage preparations from individual clones were tested for binding activity against HA of influenza A/New Caledonia/20/99 virus. The clones showing high reactivity were further analyzed for cross-reactivity against HA antigens of A/California/07/2009 (H1N1)-like or/Udorn/307/72(H3N2) viruses by phage ELISA as previously described (25). Briefly, of HA of each of the virus diluted in coating buffer were applied to ELISA plate wells and incubated overnight at 4°C. The wells were washed twice with 1× PBS and blocked with 2% SMP (2% skim milk powder in PBS) for 2 h at RT. HA-specific precipitated recombinant phage preparation (1:2 diluted in 2% SMP) was added to each well and incubated at RT for 1 h. After washing six times with 400 μl PBS per well, the bound phage were detected with the anti-M13 antibody B62-FE2 against PVIII coat protein of the M13 phage.

### Peptide ELISA

Five peptides (27) were commercially synthesized and used for testing the phage-scFv antibodies for analysis of the epitopes identified by them by peptide ELISA. The peptide sequences were chosen based on the previous report that these were linear HA epitopes and relatively conserved among different sub-types of influenza A viruses. The peptides (about 2 μg/well) in a final volume of 50 μl coating buffer (Na_2_CO_3_⋅Na_2_HCO_3_, pH 9.6) were coated onto ELISA wells and incubated overnight at 4°C. The wells were washed with PBST (1× PBS with 0.1% Tween 20) once, followed by three washes with PBS. The wells were incubated with blocking buffer (1% BSA in 1× PBS) at 37°C for 1 h. After washing, selected phage-bound scFv was applied onto each well. After incubation at 37°C for 2 h, the wells were washed with washing buffer (1M NaCl, 0.05% Tween 20) for four times, and incubated with anti-mouse HRP conjugated antibody (1:1000) for 1 h followed by one washing with PBST and three washings with PBS and detection with substrate, and absorbance measurement at 490 nm in ELISA reader. Sequences of the peptides tested against the scFv antibodies are mentioned in Table 1.

**Table 1:** **Sequences of the peptides tested against the scFv antibodies**.

Peptide	Position	Sequence
P1	38–52	EKNVTVTHSVNLLED
P2	58–72	LCKLRGVAPLHLGKC
P3	318–332	GKCPKYVKSTKLRLA
P4	318–332	GACPRYVKSNTLKLA
P5	318–332	GECPKYVRSAKLRMV

### ELISA for diagnosis of influenza A virus infection

A total of 53 human nasal/throat swab specimens were tested for development of the phage-scFv antibody-based diagnostic ELISA test. The samples were collected from various hospitals in Delhi, India and were already tested with the standard real time RT–PCR test. A total of 53 samples were tested. Forty-seven samples were from patients, who acquired natural infection of influenza A virus, of which 10 patients were diagnosed for pH1N1/09 virus infection and 37 for seasonal influenza A virus (14 H1N1 + 23 H3N2) infection by real time RT–PCR. Six individuals served as healthy controls that did not encounter any influenza virus infection since more than 6 months from the date of sample collection, and were also real time RT–PCR negative for the viral infection.

The ELISA wells were coated with 1:2 diluted clinical specimens in coating buffer overnight at 4°C, followed by washing with PBST (1× PBS with 0.05% Tween20) and PBS. The wells were blocked with 2% skimmed milk in PBST for 1 h at 37°C and incubated with C5 phage-scFv antibody at 37°C for 2 h. The wells were washed with PBST and PBS followed by detection with the anti-M13 phage antibody B62-FE2 against PVIII coat protein of the M13 phage as described before. Following the addition of TMB substrate, the reaction was stopped and the absorbance was measured at 450 nm in an ELISA plate reader. Each sample was tested in duplicate and the maximum absorbance was measured by using the P3 peptide in place of the test samples. The samples from healthy controls served as negative controls for the test. The sensitivity and specificity of the test (26) were calculated by the following formulae, where determination of the true positive or true negative samples was done by real time RT–PCR analysis.

$$\begin{array}{llll} \text{Sensitivity}\;= & \text{[True positive divided by sum of true positive and false negative]}\times 100\; & & \;\\ \text{Specificity}\;= & \text{[True negative divided by sum of true negative and false positive]}\times 100\; & & \; \end{array}$$

Results were expressed as sensitivity and specificity of the test in comparison to real time RT–PCR test for diagnosis of influenza A virus infection.

## Results

**Figure 1:**
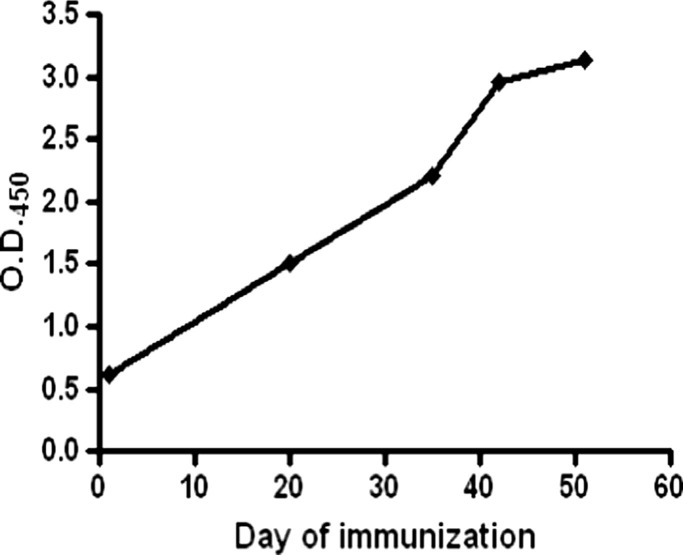
**Graphical representation of the mean antibody titers in sera of immunized mice before each immunization**. High anti-HA antibody levels were detected in the hyper-immunized animals after 51 days of the immunization schedule.

A total of 6- to -11-week-old Balb/c mice were immunized via intra-muscular route with pandemic influenza A H1N1 (2009) virus. A relatively high immune response was observed after booster doses of pandemic influenza A H1N1 (2009) virus as confirmed by ELISA with immunized mice sera. The absorbance measured for the anti-HA antibodies before the first virus administration was 0.620, before fifth immunization dose was 3.129 (Table 2) and exceeded the detectable range post 48 h of the fifth immunization. The increase in the antibody titer upon each immunization is represented in the Figure 1.

**Table 2:** **Absorbance values observed in ELISA for estimation of anti-HA antibodies in mice sera, after each immunization with influenza A/New Caledonia/20/99(H1N1) virus**.

S.No.	Day	OD_450_
1	1	0.620
2	20	1.507
3	35	2.214
4	42	2.956
5	51	3.129

### Amplification and cloning of the antibody genes

**Figure 2:**
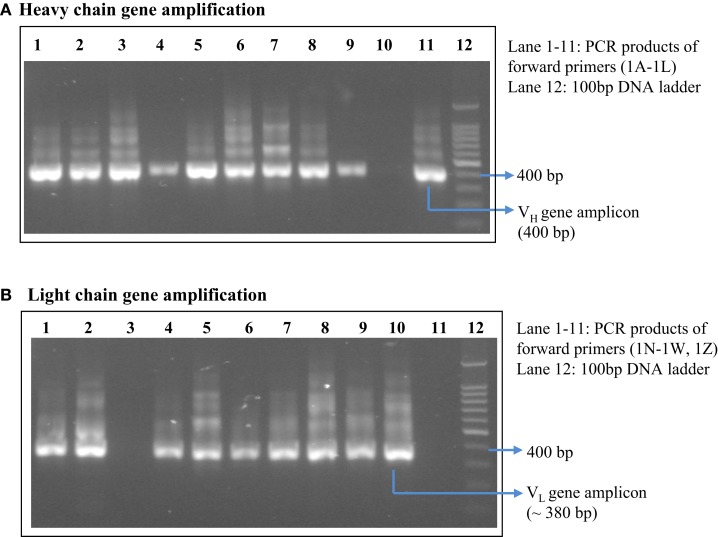
**Amplified light chain DNA (~380 bp) (B) and heavy chain DNA (400 bp) (A) using mouse IgG library primer set**. Lanes 1–11 represent PCR products from 11 different degenerate forward primers supplied with the kit.

**Figure 3:**
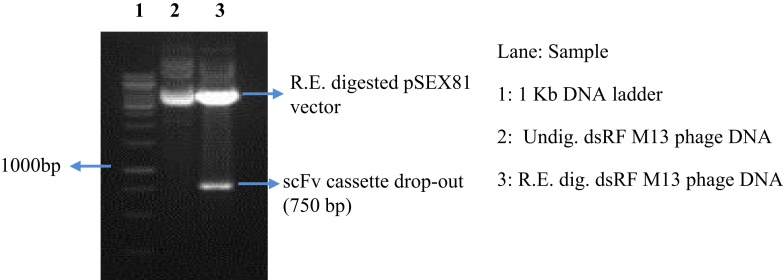
**Cloning of the complete scFv cassette in the pSEX81 phagemid vector**. The phage-display library of the murine scFv gene pool was constructed by insertion of the antibody light and heavy chain gene DNA into the phagemid pSEX81 vector. Lane 3 represents the drop-out of the scFv DNA upon restriction digestion with the restriction enzymes, *Nco*I and *Not*I, flanking the scFv cassette.

After achieving a considerable antibody titer against the HA antigen, spleen was collected from two of the hyper-immunized mice. A total of 7.5 × 10^6^ viable B cells were obtained after preparation of single cell suspension of hyper-immunized mice’s spleen. Three and a half million B cells were used for total RNA isolation. One microgram of the mRNA, which was isolated from the total cellular RNA, was subjected to synthesis of cDNA to be utilized as template for amplification of the antibody genes. The V_L_ and V_H_ genes were individually amplified using a commercially available mouse IgG primer set. DNA bands at expected sizes (as mentioned in the manufacturer’s protocol) of 400 bp for V_H_ and ~380 bp for V_L_ were observed in 10 out of 11 degenerate forward primers for V_H_ gene DNA and 9 out of 11 primers for V_L_ gene DNA (Figure 2). All the amplicons of V_L_ and V_H_ gene were individually purified from the gel. First, the V_L_ gene pool was cloned in the pSEX81 phagemid vector followed by cloning of the V_H_ gene pool. Digestion with the restriction enzymes flanking the scFv cassette (*Nco*I and *Not*I) confirmed the insertion of both the genes and thus cloning of the complete scFv pool in the phagemid vector (Figure 3).

### Phage rescue

The scFv displaying recombinant phages were rescued from pSEX81-scFv transformed *E. coli* XL1-Blue cells after infection with the hyperphage (M13K07ΔPIII). After overnight incubation at 34°C/220 rpm, the culture showed uniform turbidity. Different dilutions of the precipitated phage preparation were titrated against various dilutions of the tracing antibody for optimization. A dilution of 1:2 of the rescued phage and 1:200 of the tracing antibody were found optimum for detection of the recombinant phages in ELISA.

**Figure 4:**
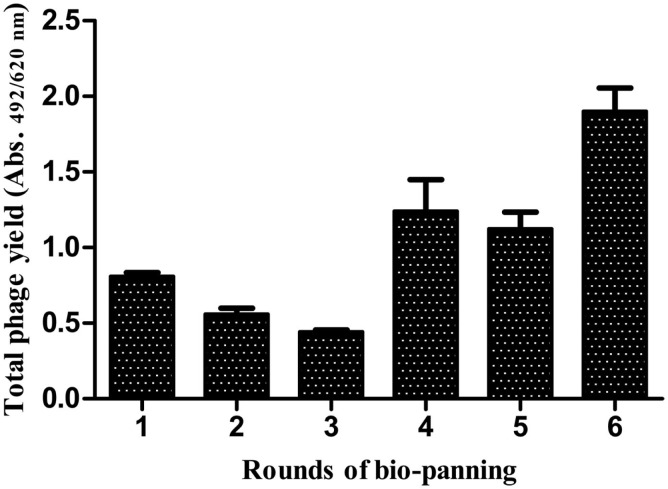
**Affinity selection of phage-bound anti-HA scFv antibodies from the antibody library**. A considerable rise in the specific scFv antibodies was observed after the sixth round of bio-panning against the A/New Caledonia/20/99(H1N1) virus.

The HA-specific recombinant phages were selected by the bio-panning procedure. The phage yield was observed to show a marked increase after the sixth round of bio-panning (Figure 4) against the influenza A/New Caledonia/20/99 virus strain.

### Cross-reactivity and peptide ELISA

**Figure 5:**
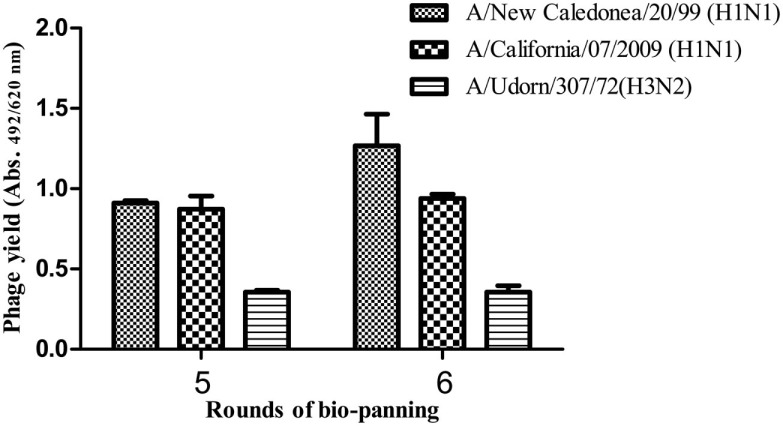
**Binding activity of the polyclonal rAb preparation against the three viral strains**. The total phage yield after the last two rounds of bio-panning was analyzed against the A/New Caledonia/20/99(H1N1), A/California/07/2009(H1N1)-like and A/Udorn/307/72(H3N2) viruses by ELISA.

**Figure 6:**
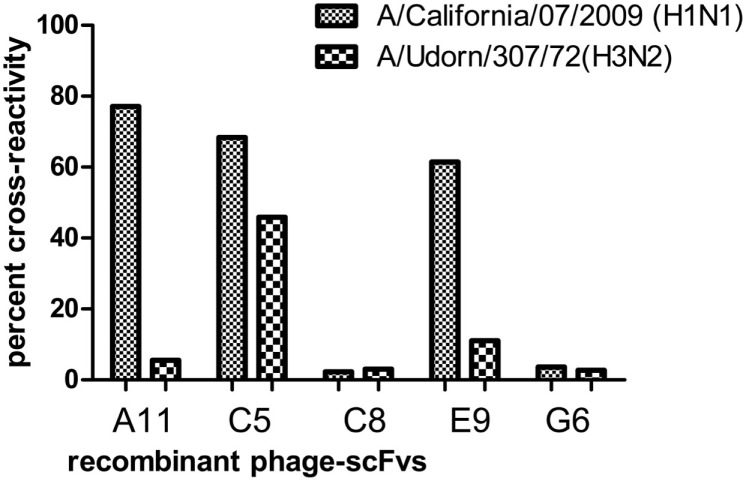
**Assessment of cross-reactivity of the relatively high affinity anti-HA (A/New Caledonia/20/99(H1N1) virus) monoclonal rAbs against the other influenza A virus sub-types, A/California/07/2009 (H1N1)-like and A/Udorn/307/72(H3N2) viruses**. The ELISA absorbance value for each of the cross-reactive strains was divided by that of the specific strain and multiplied by 100 to generate the percent cross-reactivity.

**Figure 7:**
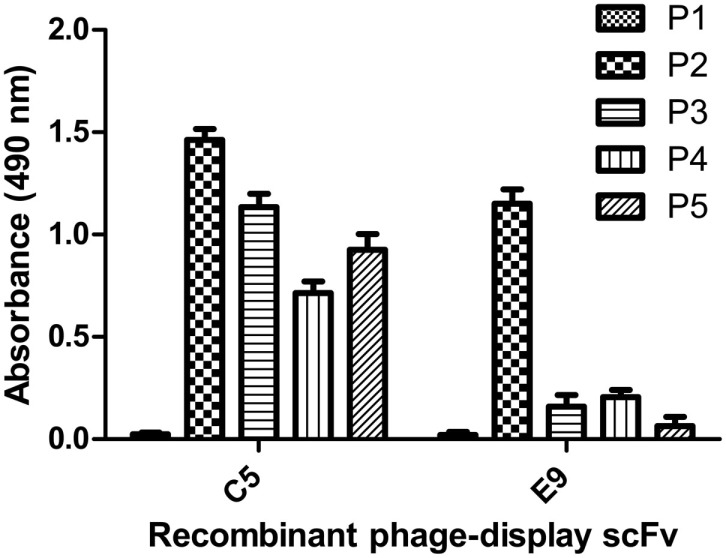
**Peptide ELISA**. Different peptide sequences (representing linear HA epitopes) were synthesized and tested against the monoclonal phage-display antibodies for analysis of the binding activity. The C5 antibody, which was found to react with all the four HA peptides was used for development of the diagnostic ELISA test.

The influenza A/New Caledonia/20/99-bio-panned phage preparation was tested against the pandemic H1N1/09 and seasonal H3N2 viruses, after fifth and sixth rounds of bio-panning and it was observed that there was no considerable increase in the total phage yield against the non-specific viral strains, although the reactivity levels of the recombinant scFv-phage preparation, after the fifth round, were similar against the two H1 sub-types (Figure 5). Of the 56 clones, five (A11, C5, C8, E9 and G6) showed high reactivity against the A/New Caledonia/20/99 (data not shown). Of these five scFv clones, three clones (A11, C5 and E9) cross-reacted well with A/California/07/2009 (H1N1)-like virus, and one (C5) cross-reacted with A/Udorn/307/72(H3N2) virus (Figure 6).The high reactivity rAb clones were further analyzed by peptide ELISA. The identification of the epitopes, being recognized by the scFv clones was done against the previously characterized peptides (27). The phage-scFv antibodies, A11, C8 and G6, did not bind to any of the peptides tested, while C5 and E9 showed binding activities with the peptide P2, and C5 phage-scFv antibody showed variable binding activities with three peptides P3, P4, and P5 (Figure 7). The peptide P1 was not recognized by any of the two phage-scFv anti-HA rAbs.

### ELISA for diagnosis of influenza A virus infection

The ELISA test was developed using the C5 phage-scFv antibody, as it was found to react with all the three HA, i.e., seasonal H1, pandemic H1, and H3 antigens. The peptide P3 was taken as the positive control and as a measure of maximum absorbance. The mean absorbance value of the negative specimens was taken as cut-off to determine the sample positivity. Among the 47 true positive specimens, the rAb-based ELISA test detected influenza A virus in 38 samples, while all the six true negative controls were observed to be negative by the ELISA test. Thus, the ELISA test developed herein showed 83.9% sensitivity and 100% specificity.

## Discussion

Influenza A viruses pose considerable economic burden both on the society and individuals in terms of consumption of health care resources and lost productivity. The occurrence of frequent flu outbreaks leads to large-scale socio-economic losses and calls for improvement in the production of reagents, in terms of large scale production, uniform quality, low cost, and stability for effective diagnosis and management of the disease. With the advent of upgraded technologies, rAbs have provided great advantages against the conventional monoclonal antibodies (28, 29). Once developed, rAbs are economically one of the most feasible diagnostic and therapeutic agents and allow mass scale production. A rAb can be developed by various procedures, for e.g., by conventional recombinant expression in prokaryotic expression system or by recombinant methods, such as, phage-display technology. Phage-display technology is a powerful technique (30), which allows easier and faster production of antibodies. The resulting molecules exhibit high antigen specificity and can be produced in large quantities, thus avoiding the use of conventional time-consuming methods like hybridoma technology. Therefore, in the present study, we aimed toward development of a recombinant antibody-based economical test for a cheaper yet effective diagnosis of human influenza A virus infection.

An antibody-phage-display library was constructed from B cells of Balb/c mice hyper-immunized with pandemic influenza A H1N1 (2009) virus. The V_H_ and V_L_ chain genes were amplified using commercially available degenerate primer sets. The sequences of the mouse IgG library primers were finalized after analysis of the various antibody sequences available in the Kabat-Wu data book (31), so as to enable amplification of as much possible antibody variable regions. Since, the desired template sequences were not known; similar sequences were grouped for choosing putative primer sequences from each group, which were then compared against all database sequences for selection of best-fitting primer sequences. The process was repeated until all the database sequences were covered (31).

In order to diminish a bias against particular sequences, the amplification of V_H_ and V_L_ genes was done by two polymerase chain reactions using two separate mouse IgG library primer sets. The first set provided an unbiased amplification, while the second set was necessary for introduction of restriction endonucleases sites to allow cloning of the amplified immunoglobulin gene fragments into pSEX81 phagemid vector. The recognition sequences of *Nco*I (5′) and *Hin*dIII (3′) were added to the amplified heavy chain gene fragments and *Mlu*I (5′) and *Not*I (3′) to the amplified light chain gene fragments. The choice of restriction enzymes was based on various advantages offered by them, viz. (i) low probability to cut within mouse V_H_ or V_L_ coding regions, (ii) optimal cloning efficiency by production of overlaps of four nucleotides or more, (iii) methylation independence, and (iv) more than 90% efficiency in double digestion reactions (32).

DNA bands at expected sizes of ~400 and ~380 bp were observed after PCR amplification of V_H_ and V_L_ genes, respectively, which were individually purified for cloning in pSEX81 phagemid vector. The complete scFv cassette (750 bp) containing phagemid clones were transformed in *E. coli* XL1-Blue cells and subjected to rescue of recombinant phages expressing the pIII-scFv fusion proteins on their surface. The rescued phage antibody library was subjected to selection of the anti-HA antibodies by bio-panning, which is an affinity-based selection process. The antibody with the desired specificity is selected from a recombinant heterogeneous antibody repertoire and enriched over a million fold by selection against the specific antigen (33). The rescued phages were quantified after each round of bio-panning and a marked increase was observed after sixth round of bio-panning. The recombinant scFv-displaying phage preparations, bio-panned against the influenza A/Caledonia/20/99 virus, were analyzed for cross-reactivity after fifth and sixth rounds against the A/California/07/2009 (H1N1)-like or A/Udorn/307/72(H3N2) viruses. We observed that the recombinant phages reacted to similar levels against the H1N1 strains, after the fifth round of bio-panning. However, after the sixth round of the affinity selection, an insignificant increase in the phage yield was observed against the pH1N1/2009 virus. The percentage of cross-reactivity of the recombinant phages for the H3N2 strain of the virus was very low (~40%), which can be explained by the dissimilarity of the H3 from the H1 sub-type. We chose to continue our study with the phage-bound antibodies, as the conversion of scFv-phage antibody to its soluble form leads to alteration in the antibody specificity (34). Further analysis of the single phage-scFv clones showed that six clones had relatively high reactivity against the specific influenza virus sub-type (A/Caledonia/20/99 virus), of which the six clones (A11, C5 and E9) and one clone (C5) showed cross-reactivity with A/California/07/2009(H1N1)-like virus, and A/Udorn/307/72(H3N2) virus respectively. Since, the clones were selected via a rigorous procedure of affinity selection and showed cross-reactivity among the other HA sub-types, we attempted to test the clones against some of the already validated conserved linear epitopes (27). The C5 scFv-phage clone was found to react with three different peptides corresponding to three different HA sub-types, with variable reactivity, though. Therefore, the C5 scFv-phage antibody was employed to develop an ELISA test for diagnosis of the human influenza A virus infection in clinical specimens. The non-reactivity of the other three phage-scFv rAbs, i.e., A11, C8, and G6 antibodies, may be due to their specificities against the epitopes other than the ones tested in the experiment. Moreover, in peptide ELISA, only linear epitopes could be tested, therefore, it is likely that A11, C8, or G6 would be specific against conformational epitopes. The most widely and the recommended real time RT–PCR test imposes restriction due to its high cost, however, the ELISA test proposed in the current study may be performed in any laboratory with basic facilities/manpower/expertise. The sample size tested in the current study showed 83.9% sensitivity and 100% specificity for the ELISA test, however, a higher sensitivity is crucial for application of the test for detection of influenza A virus in the field specimens. In this regard, the work is in progress in our laboratory by testing of higher number of routinely collected human clinical samples.

## Conclusion

Presently, the real time RT–PCR test is the most widely used technique to diagnose flu (6). However, in the developing countries, the high cost of such test poses limitations in better management and efficient surveillance of the disease. Therefore, we attempted to develop the diagnostic ELISA test as a low cost alternative for the diagnosis of influenza virus infections. The number of samples tested in the present study yielded 83.9% sensitivity and 100% specificity of the test. However, further validation of the results by testing a larger number of samples would be required, which would further help in determination of the efficiency of the ELISA test. Following such validation, the ELISA test developed by us holds significance, as it can be used as an initial screening method for the diagnosis of influenza virus infection in human specimens.

## Conflict of Interest Statement

The authors declare that the research was conducted in the absence of any commercial or financial relationships that could be construed as a potential conflict of interest.
